# Divalent metal content in diet affects severity of manganese toxicity in *Drosophila*

**DOI:** 10.1242/bio.060204

**Published:** 2024-01-05

**Authors:** Zahraa A. Ghosn, Kailynn M. Sparks, Jacob L. Spaulding, Sanjana Vutukuri, Mirza J. J. Ahmed, Mark F. A. VanBerkum

**Affiliations:** Department of Biological Sciences, Wayne State University, Detroit, MI 48202, USA

**Keywords:** Manganese, Diet, *Drosophila*, Life span, Calcium, Magnesium

## Abstract

Dysregulation of manganese (Mn) homeostasis is a contributing factor in many neuro-degenerative diseases. Adult *Drosophila* are sensitive to excessive levels of dietary Mn, dying relatively early, and exhibiting biochemical and mobility changes reminiscent of Parkinsonian conditions. To further study Mn homeostasis in *Drosophila*, we sought to test lower levels of dietary Mn (5 mM) and noted a striking difference in Canton-S adult survivorship on different food. On a cornmeal diet, Mn-treated flies live only about half as long as untreated siblings. Yet, with the same Mn concentration in a molasses diet, adults survive about 80% as long as untreated siblings, and adults raised on a sucrose–yeast diet are completely insensitive to this low dose of dietary Mn. By manipulating metal ion content in the cornmeal diet, and measuring the metal content in each diet, we traced the difference in lifespan to the levels of calcium and magnesium in the food, suggesting that these ions are involved in Mn uptake and/or use. Based on these findings, it is recommended that the total dietary load of metal ions be considered when assessing Mn toxicity.

## Teaser

**Summary:** In a series of life span survival assays on different diets, this study shows that other divalent metals in the diet, especially calcium and magnesium, protect adult *Drosophila* from manganese toxicity.

## INTRODUCTION

Manganese (Mn) is an essential trace mineral that is used as a cofactor by many enzymes that contribute to several metabolic processes (Roth et al., 2013; O'Neal and Zheng, 2015; Tinkov et al., 2021). This includes bioenergetics, oxidative stress, protein processing and aggregation, and metal ion homeostasis. Mn accumulation leads to Manganism, which is defined by symptoms that are similar to Parkinson's disease (Kornblith et al., 2018; Guilarte and Gonzales, 2015). Both conditions are associated with alterations in Radical Oxygen Species (ROS) activity, decreased mitochondrial function, and the aggregation of alpha-synuclein proteins (Kornblith et al., 2018; Power et al., 2017). Indeed, dysregulation of metal ion homeostasis, including that of Mn, is now recognized as a potential contributing factor in several other neurodegenerative conditions, including Alzheimer's and Huntington's diseases (Lang et al., 2012; Pfalzer and Bowman, 2017; Chib and Singh, 2022; Kim et al., 2022). To help understand the dynamics of Mn homeostasis and the effects of elevated Mn in neurodegenerative conditions, it is important to continue to develop animal models (Taylor et al., 2020).

Mn homeostasis is complex, involving interactions with processes regulating the levels of other divalent metals (Roth et al., 2013; Balachandran et al., 2020; Tinkov et al., 2021). Mn and iron (Fe), for instance, share similar size and redox potential, and both occur as physiologically relevant divalent and trivalent species in the body (Roth et al., 2013). Mn is also transported by transferrin and uses membrane transporters in common with Fe, including the Divalent metal transporter-1 (DMT-1), which is a major candidate for Fe and Mn absorption in the gastrointestinal tract and across the blood brain barrier (Shawki et al., 2012; Illing et al., 2012; Mackenzie et al., 2010). Mn is also carried into cells by transporters of the SLC38A family of zinc transporters (Zip), including Zip8 and Zip14, the latter of which is expressed in the small intestine and important for dietary Mn absorption (Pinilla-Tenas et al., 2011; Wang et al., 2012; Dechen et al., 2015; Qin et al., 2013; Winslow et al., 2020). Members of the Zinc transporter (ZnT) family move metals, including Mn, zinc and iron out of cells, or into the Golgi or Endoplasmic reticulum for removal via the secretory pathway (Levy et al., 2019; Wang et al., 2009, 2020; Gurol et al., 2022; 2023; Nishito et al., 2016; Zogzas and Mukhopadhyay, 2018). Shuttling of Mn to the secretory pathway may also use store-operated Ca^2+^ channels, including the Sarco(Endo)plasmic Reticulum Ca^2+^-ATPase (SERCA) or the secretory pathway Ca^2+^/Mn^2+^ ATPase (SPCA; Sepulveda et al., 2012; Kambe et al., 2016). In the Golgi, Mn is involved in protein glycosylation (Kaufman et al., 1994; Kambe et al., 2016; Sepulveda et al., 2012). As such, when in dietary excess, the absorption, use and excretion of Mn can alter metabolic and secretory pathways, while also disrupting the homeostatic regulation of other trace metals. In combination, these processes are contributing factors to pathophysiology and neurodegenerative conditions (Winslow et al., 2020).

The fruit fly, *Drosophila melanogaster*, is highly susceptible to elevated dietary Mn and exhibits many of the features observed in rodent and *C. elegans* models of Mn toxicity (Gubert et al., 2018; Balachandran et al., 2020; Pfalzer et al., 2020; Pankau and Cooper, 2022). With an increased dietary load, Mn levels are elevated in both the whole body and the heads of treated flies; a preferential loss of dopaminergic neurons has also been detected (Bonilla-Ramirez et al., 2011; Mohandas et al., 2017). Adult flies treated with high levels of dietary Mn also exhibit shortened life span, decreased mobility, and anti-geotactic climbing defects. At the physiological level, several biochemical markers, including tyrosine hydroxylase activity, dopamine levels, and mitochondrial activity, are affected (Mohandas et al., 2017; Silva et al., 2021). A recent study identified acute effects of Mn toxicity, including altered neurotransmission at the *Drosophila* neuromuscular junction (a glutamate synapse) and abnormal cardiac function (Pankau et al., 2022). Together, these outcomes identify *Drosophila* as a useful model organism to study Mn toxicity.

Most *Drosophila* studies focused on the effects of high Mn doses that severely shortens adult fly life span, often to less than 20 days. That is, flies are dying at relatively young ages, as under ideal culture conditions adults can survive over 70 days (Ormerod et al., 2017). Given the late onset of many neurodegenerative conditions, it seems advisable to develop rearing conditions that allow animals to live longer under elevated Mn exposure. Looking at the role of diet (Ormerod et al., 2017), sugar and yeast content both impact lifespan in *Drosophila*, regardless of caloric intake, with high yeast content reducing overall food intake, and elevated carbohydrates increasing feeding behaviors (Skorupa et al., 2008). Other macronutrient content, including cholesterol, amino acids, and vitamins can also modulate lifespan (Grandison et al., 2009a,b). In the above *Drosophila* studies of Mn toxicity, a number of different diets have been used, making it difficult to ascertain how diets effect Mn toxicity.

Bonilla-Ramirez et al. (2011) treated female flies with 0.5- or 1-mM Mn in a 1% glucose solution and observed a significant decline in survivorship within approximately 15 days. More recently, Silva et al. (2021) used a mixed population (male and female) of flies on a hardy cornmeal-based diet with milk powder as a protein source. They observed a decline in survivorship within 10 days and a corresponding decrease in both geotaxis and exploratory mobility even at 3- or 5- mM dietary Mn. The protein content may be particularly important, as adding whey to a standard wheat germ diet attenuated the effect of Mn toxicity (Mohandas et al., 2017). Other dietary supplements also attenuate the effects of manganese treatment, including gamma-oryzanol, diphenyl diselenide, and an alkaloid leaf extract (Mohandas et al., 2017; Silva et al., 2021; Adedara et al., 2016; Oboh et al., 2018). How these supplements alter Mn toxicity remains unclear, but they may affect Mn availability or counteract the putative changes Mn causes in cell metabolism (see Bonilla et al., 2012, and Kreutzmann et al., 2012). Nevertheless, available data support the idea that the diet affects the degree of Mn toxicity and general life span. To better understand the relationship between Mn homeostasis and neurodegenerative conditions, the present study set out to standardize the diet using lower doses of Mn with the goal of increasing the life span of exposed adult flies. Comparing the effects of two common fly diets, we report a dramatic effect of diet on adult sensitivity to low levels of dietary Mn, and systematically uncover a role for dietary calcium and magnesium in this disparate effect.

## RESULTS

### Adult sensitivity to Mn differs between molasses and cornmeal diets

**Fig. 1.**
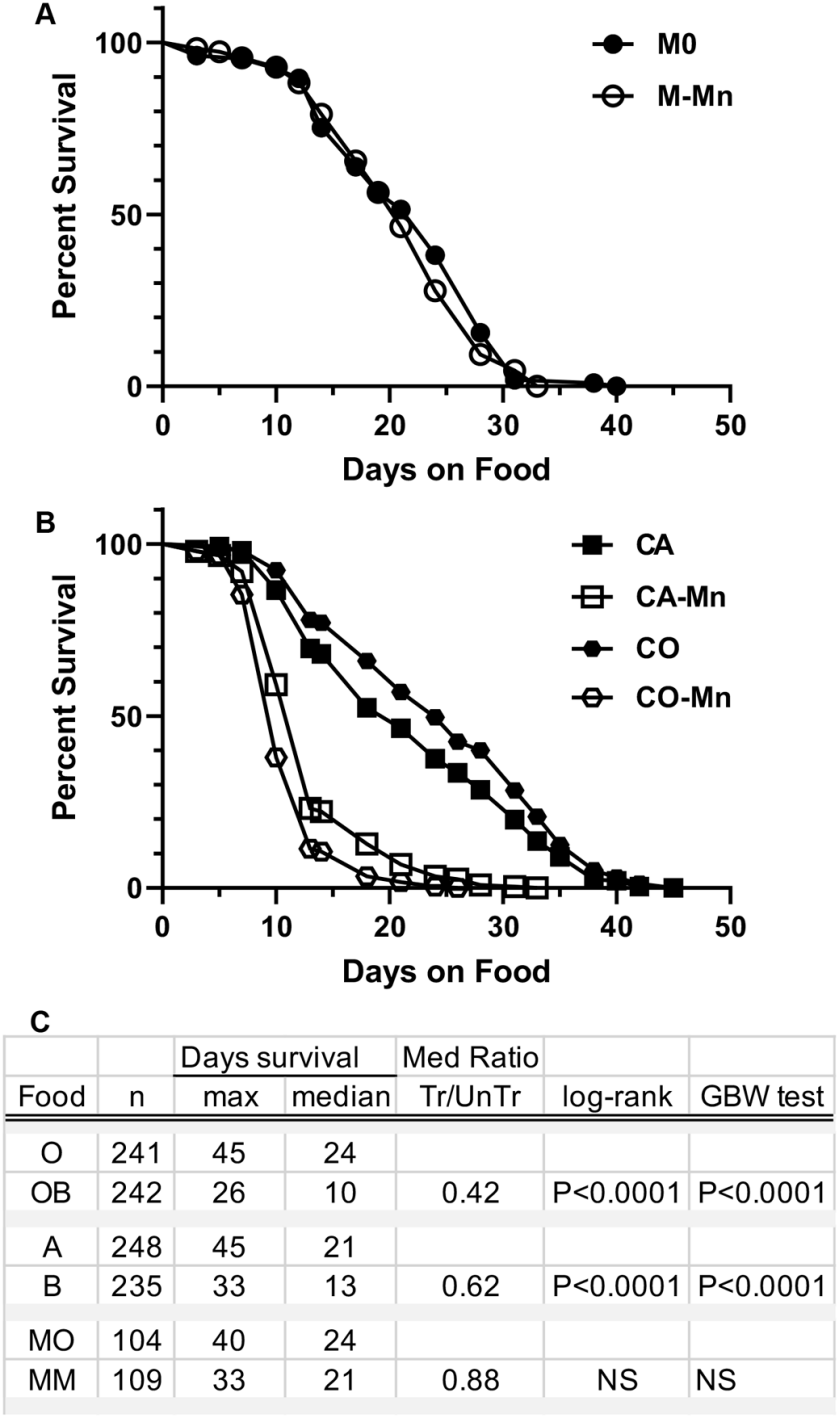
**Manganese toxicity is markedly affected by diet.** Kaplan–Meier survival curves are shown for adult Canton-S sibling flies raised on different diets with or without MnCL_2_ treatment. (A) On a metal-rich molasses diet, there is no difference in adult survivorship with (M-Mn) or without (MO) Mn-treatment. (B) On metal-poor cornmeal diets, adult survivorship on Mn-treated cornmeal diets (CO-Mn and CA-Mn) is markedly reduced compared to siblings on untreated (CO and CA) diets. (C) Tabulated data for each survivorship curve is shown by diet and includes the final number of flies assessed (*n*) combing triplicate samples. Maximum and median life spans (days) as calculated by Prizm and the treated to untreated ratio of median life span are also shown. Statistical significance is indicated for both the Mantel–Cox (MC) log rank and Gehan–Breslow–Wilcox (GBW) tests. Data on the molasses diet is a representative experiment using four replicate vials, with similar results observed in three other smaller runs. Data on the cornmeal diets represent the combined data of duplicate sets of five vials each, although similar results were observed in three additional runs.

To search for diet conditions that would allow longer life span at low doses of dietary manganese, we generated life span curves for adult Canton-S flies on three different diets in the presence and absence of 5 mM MnCl_2_ (Fig. 1). The three diets included a commonly used molasses diet with (M-Mn) or without (MO) manganese added, and two versions of a cornmeal diet made using deionized water. The basic cornmeal food (CO) has very low levels of metal ions provided solely by the dry ingredients, while the second version (CA) was supplemented with millimolar amounts of calcium and magnesium and micromolar amounts of other trace metals as indicated in Table 1B. The corresponding Mn treated diets are designate CO-Mn and CA-Mn, respectively.

**Table 1a:**
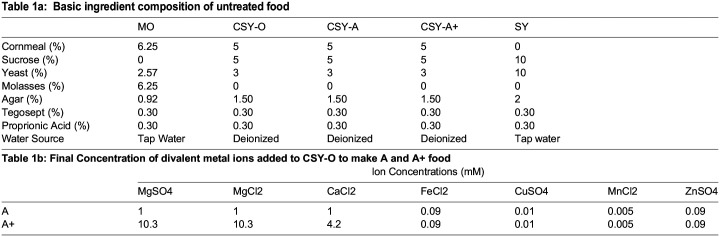
Basic ingredient composition of untreated food

**Table 1b: Final Concentration of divalent metal ions added to CSY-O to make A and A+ food**							
	Ion Concentrations (mM)						
	MgSO4	MgCl2	CaCl2	FeCl2	CuSO4	MnCl2	ZnSO4
A	1	1	1	0.09	0.01	0.005	0.09
A+	10.3	10.3	4.2	0.09	0.01	0.005	0.09

*Drosophila* Canton-S adults exhibited similar life span curves on all three diets (Fig. 1), with median and maximal survival of approximately 23 days and 40 days, respectively. On molasses food, addition of Mn (M-Mn) does not significantly shorten life span, with a treated to untreated ratio of median life span ratio of 0.88 (Fig. 1). On cornmeal diets, however, Mn-treated flies survived only about half as well as untreated flies [*P*<0.0001, Mantel­–Cox (MC) log rank and Gehan–Breslow–Wilcox (GBW) tests], corresponding to a treated to untreated ratios of median life span of 0.42 (CO-Mn/CO) and 0.62 (CA-Mn/CA), respectively (Fig. 1C). These contrasting survival data provide compelling evidence that the composition of the diets affects Mn toxicity.

### Both calcium and magnesium attenuate Mn toxicity in cornmeal diet

**Fig. 2.**
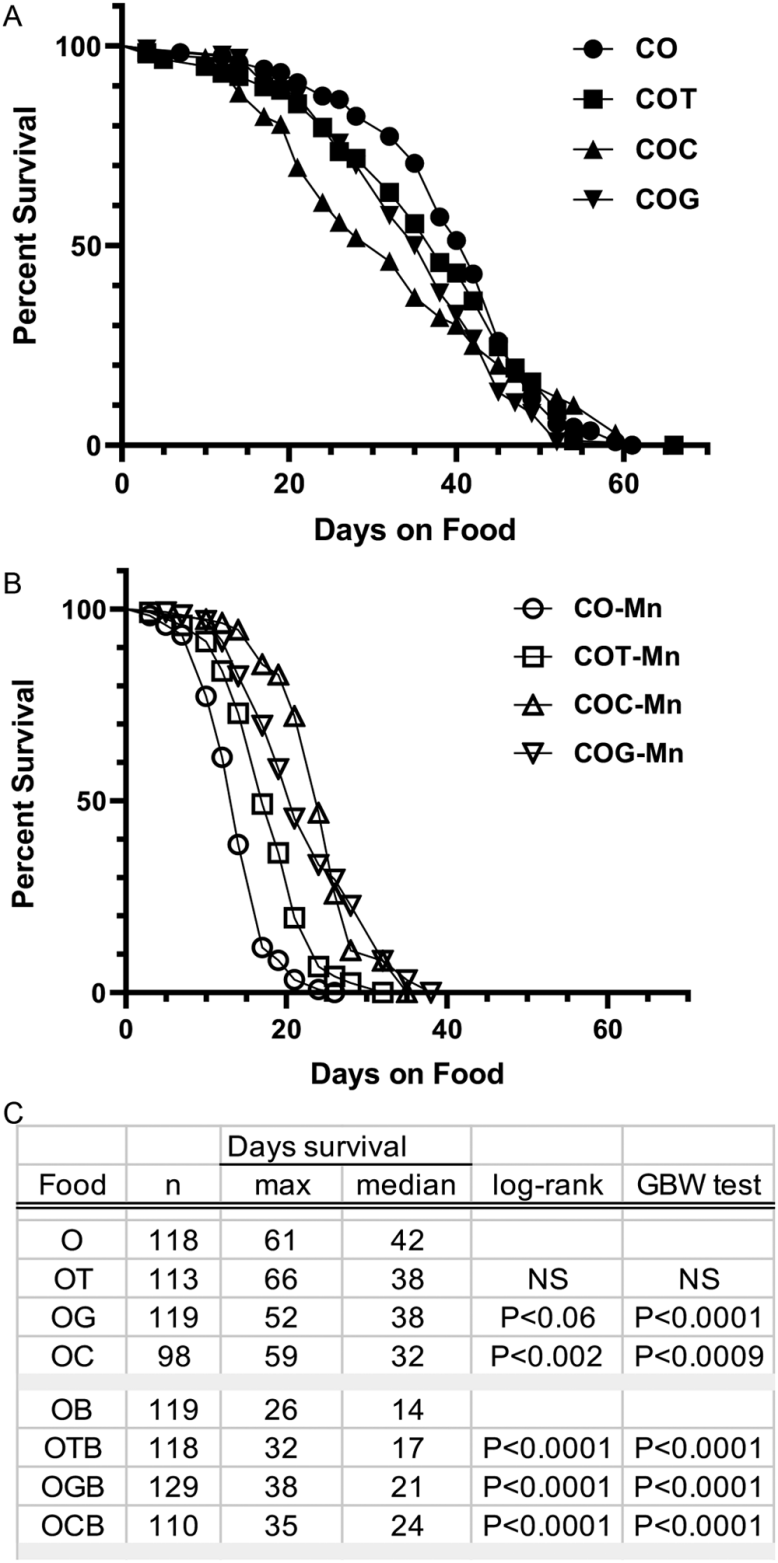
**Adding selected metals to the basic cornmeal diet (CO) reveals the importance of dietary calcium and magnesium to adult fly life span and manganese resistance.** Kaplan–Meier survival curves are shown for sibling adult CS flies raised on a series of basic cornmeal diets supplement with different metal ions. Deceased flies were counted three times a week. (A) Comparison of life span of adults raised on the basic cornmeal diet (CO) with its low metal ion content, or on this diet with only trace metals added (COT), calcium added (COC) or magnesium added (COG). (B) Comparison of the Mn-toxicity on flies raised on the same series of basic cornmeal diets as shown in A now supplemented with 5 mM MnCl_2_. (C) Tabulated data for each survivorship curves is listed by diet and includes the final number of flies assessed (*n*). Maximum and median life spans (days) as calculated by Prizm are also presented. Statistical significance is indicated for both the Mantel–Cox (MC) log rank and Gehan–Breslow–Wilcox (GBW) tests with each treatment compared to the baseline cornmeal diet, CO, or CO-Mn. Data represent a single run of five vials per food type.

We suspected that the metal ion content would be a major factor affecting life span, because the cornmeal diets were made using deionized water, and molasses, a byproduct of refining sugarcane, is loaded with ions (see Supplementary file 1 nutrition fact sheet). To address the potential effects of metal ions, we next assessed how life span and Mn toxicity of adults change as different metal ions are added to the basic cornmeal (CO) diet. That is, we analyzed the life spans of flies cultured on a series of modified cornmeal diets by making the basic cornmeal diet (CO) and then adding only CaCl_2_ (COC food), or both MgCl_2_ & MgSO_4_ species (COG food), or adding only the combination of trace metals (COT food), and compared adult life span on these foods and their Mn treated (5 mM) counterparts, COC-Mn, COG-Mn and COT-Mn, respectively (Fig. 2).

Addition of a single metal to the basic cornmeal CO diet significantly decreased the median life spans of Canton-S adult flies (Fig. 2A) (*P*<0.0001 for both statistical tests). The addition of only trace metals (COT) or only magnesium (COG) shifted the median life span from 42 days for CO food to 38 days, with the magnesium but not trace metal supplement causing a significant difference (*P*<0.002 for MC log rank test and *P*<0.0009 for GBW) (Fig. 2C). Adding only calcium ions (COC) altered the shape of the life span curve, with a stronger decrease in the early stages and a shift in median life span to 32 days (*P*=0.06 log rank test and *P*<0.0001 for GBW test) (Fig. 2C). Together, these data indicated that the composition of divalent metals in the diet affects life span, with calcium and magnesium playing the largest roles.

We further observed that calcium had the largest effect in protecting flies from manganese treatment (Fig. 2B). In the presence of Mn, adding any of the different divalent metals to the basic cornmeal diet (CO–Mn) significantly improved life spans (*P*<0.0001 for both MC log and the GBW tests), with the trace metal combination shifting median life span from 14 to 17 days, magnesium to 21 days, and calcium shifting median life span to 24 days. With the addition of calcium, a more pronounced decay phase also improves the shape of the survival curve. Taken together, these findings revealed that in the basic cornmeal diet, adding metal ions, especially calcium and then magnesium provide a significant decrease in the sensitivity of *Drosophila* to dietary manganese.

### Molasses diet has higher levels of calcium and magnesium

To quantify the total divalent metal ion content in each diet, thus accounting for dry ingredients and water source, we used inductively coupled plasma mass spectrometry (ICP-MS) (Table 2). Interestingly, compared to the basic cornmeal food (CO), the molasses (MO) food was significantly richer in calcium (∼5X) and magnesium (∼2.6×), and varying amounts of additional trace metals. One exception was zinc, which was slightly higher in the basic cornmeal food (CO). Similarly, even after adding small amounts of metals to the cornmeal diet (CA), calcium and magnesium levels remain significantly lower than in the molasses diet. Interestingly, the elevated levels of calcium and magnesium measured in the molasses food is consistent with our observations in Fig. 2, where the addition of either of these two metals significantly improves Mn resistance on the basic cornmeal (CO) food. Together, these findings led us to hypothesize that elevating calcium and magnesium in the cornmeal diet would significantly decrease Mn toxicity.

**Table 2.**
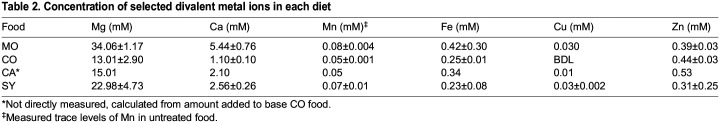
Concentration of selected divalent metal ions in each diet

### Elevating calcium and magnesium in the cornmeal diet protects flies from Mn toxicity

**Fig. 3.**
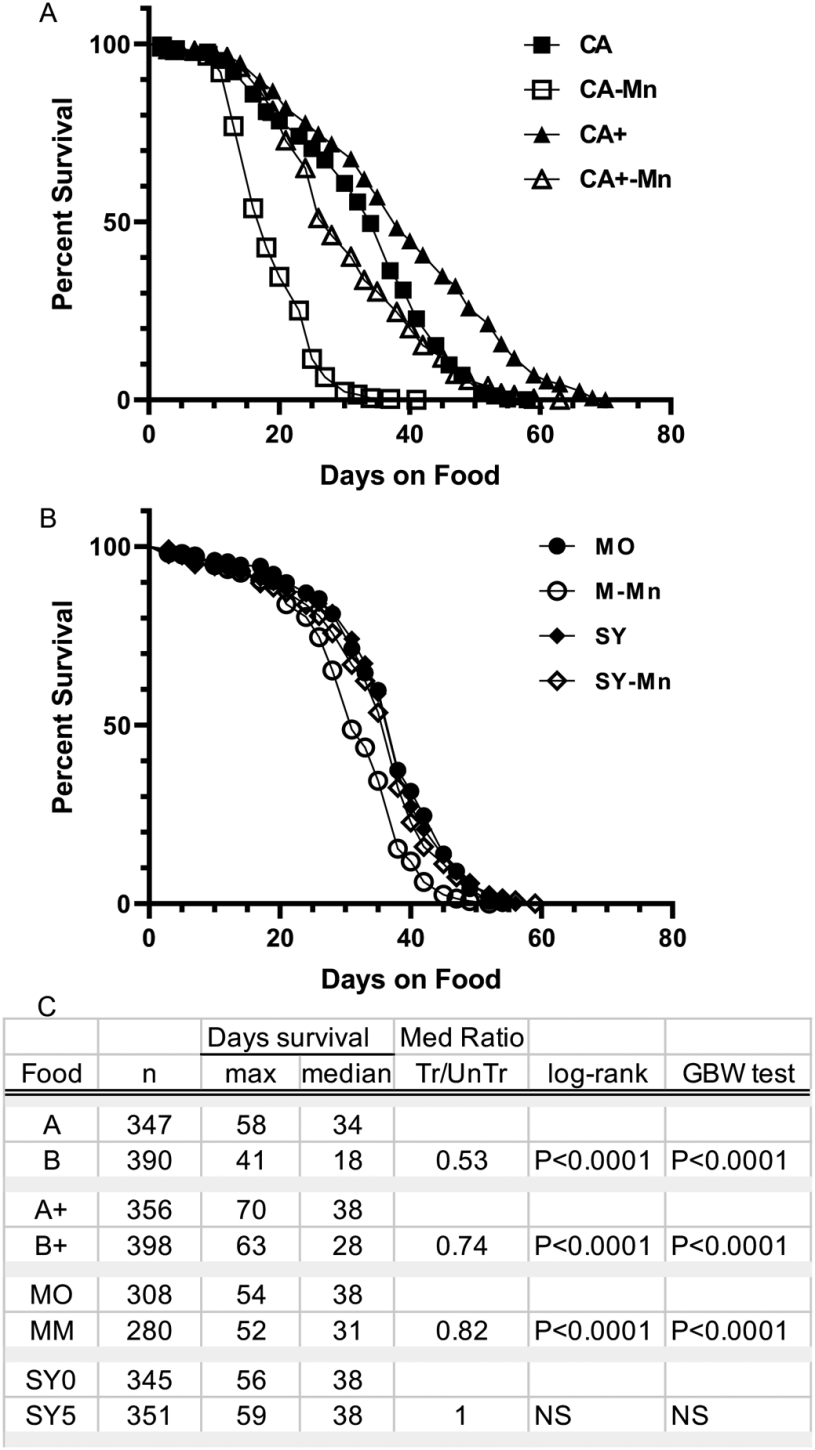
**Elevating calcium and magnesium levels in the basic cornmeal diet significantly improves resistance to Mn treatment.** Kaplan–Meier survival curves are shown for adult CS flies raised on four different sets of Mn-treated and untreated diets. Deceased flies were counted three times a week. (A) Comparison of life span on the original cornmeal CA and enriched CA+ foods demonstrates that elevated calcium and magnesium improves resistance to dietary Mn (CA-Mn and CA+-Mn). (B) Life span of flies on Mn-treated molasses (M-Mn) is slightly reduced compared to untreated siblings (MO). But adults are resistant to Mn on the sucrose-yeast diets (SY and SY-Mn). (C) Tabulated data for all survivorship curve is shown by diet and includes the final number of flies assessed (*n*), combining triplicate sets. Maximum and median life spans (days) as calculated by Prizm, and the treated to untreated ratio of median life span are also shown. Statistical significance between treated and untreated conditions is indicated for both the Mantel–Cox (MC) log rank and Gehan–Breslow–Wilcox (GBW) tests. Data for the cornmeal and SY diets represent three replicates of five vials each, and for the molasses three replicates of four vials each. Molasses and SY curves were initiated at different times but shared the same housing conditions.

To enrich the levels of calcium and magnesium in cornmeal diets, we simply added more calcium and magnesium to match the higher (+) levels determined in molasses food, while retaining the standard amount of trace metals added to CA food. We then ran survival curves, directly testing the life span of flies on this newly enriched A food (designate CA+) and its Mn-treated counterpart (CA+-Mn). For direct comparison, flies were also tested in parallel on the CA and CA-Mn diets that have the original lower levels of divalent metals. In these life span curves, to eliminate potential effects of infection, we also added antibiotics (Penn–Strep) to the media, a practice common in longevity studies (e.g. Bazzell et al., 2013; Sujkowski et al., 2022). With addition of antibiotics (Fig. 3), maximal life span increased significantly to approximately 60 and 70 days, and median survival increased to 34 and 38 days on the original CA and enriched CA+ food respectively (*P*<0.001 both MC log rank and GBW tests). While enriching for calcium and magnesium yields a small improvement on life span, we noted a significant improvement in Mn resistance. That is, Mn addition to the original CA diet continued to significantly shorten life span by about one-half (median life span ratio CA-Mn/CA=0.53), but as hypothesized, the enriched levels of calcium and magnesium in CA+ food significantly reduced manganese toxicity, with a median life span ratio of 0.74 (CA+-Mn/CA+; *P*<0.0001 for both MC log rank and GBW tests). Indeed, with Mn treatment, the median life span of 28 days observed on the enriched CA+-Mn cornmeal diet is similar to the 31 days on Mn-treated molasses (M-Mn) food (*P*=0.6 for MC log rank and *P*<0.009 for the GBW test). The different statistical tests may be highlighting the slower exponential decline and extended tail of the life span curve of adult flies living on the untreated enriched cornmeal food (CA+). On these same foods, the median life spans (38 days) of untreated CS flies are not significantly different from controls (Fig. 3C). Together, our data provide compelling evidence that the difference in Mn toxicity between molasses and cornmeal diets was primarily an effect of the total calcium and magnesium levels in the different diets.

As an additional test, we also assessed the life span of adult CS flies on the sucrose-yeast (SY) media commonly used in the longevity studies (Sujkowski et al., 2022). On this diet, the life span curves of CS adult flies on untreated (SY) and Mn-treated (SY-Mn) diets are superimposable (*P*=0.2 log rank and *P*=0.09 for GBW test) indicating complete resistance to 5 mM dietary Mn. To complete the comparison, the total divalent metal content of SY food was also measured (Table 2). Comparing these levels to the molasses food (and thus CA+) and the cornmeal diet with added metals (CA), SY food has approximately half the amount of calcium as found in molasses food, but slightly more than the amount present in the cornmeal CA food (2.6 versus 2.1 mM). Interestingly, the magnesium levels in SY (23 mM) splits the difference between that observed in our cornmeal CA food (15 mM) and the molasses food (34 mM). Thus, in SY food, resistance to Mn-treatment continues to track with elevated levels of magnesium and especially calcium. Indeed, comparing the four diets, the sensitivity of CS flies to dietary Mn clearly diminishes as the levels of calcium and magnesium increase in the food. Future work is needed to investigate the role of these metals in regulating Mn homeostasis and toxicity. Moreover, based on these data, it seems advisable to measure the total metal ion load of diets when assessing Mn-toxicity.

## DISCUSSION

In a variety of diets, relatively high levels of Mn are well known to shorten the life span and cause mobility issues (Bonilla-Ramirez et al., 2011; Mohandas et al., 2017), although some studies indicated a slight decline in health span at lower concentrations of dietary Mn (Silva et al., 2021). Seeking to standardize the diet and use a lower dose of dietary Mn (5 mM), we detected a striking difference in adult survivorship on molasses versus cornmeal diets. With Mn in the cornmeal diet, adult flies live about half as long as siblings on untreated food, compared to almost complete survival of siblings reared on the Mn-treated molasses food. Asking why flies are highly sensitive to dietary Mn in the cornmeal food, we systematically uncovered the importance of dietary calcium and magnesium levels in protecting the organism from manganese. First, the addition of only calcium or only magnesium to the basic cornmeal food (CO) significantly increases resistance to Mn by about a third. Second, by measuring total divalent metal content in the basic cornmeal (CO) and molasses diets, we determined that the molasses food contains significantly higher levels of both calcium and magnesium compared to the cornmeal food, and on molasses, adult flies are much more resistant to Mn treatment. Third, as a functional test, and to eliminate potential metabolic effects of different foods, we determined that selectively elevating the calcium and magnesium levels in the basic cornmeal diet to the levels found in molasses food (creating CSY-A+), improves Mn resistance, allowing adults to survive 5 mM Mn as well as they do on molasses food. Thus, on the cornmeal diet, the fly's resistance to dietary Mn is directly linked to the elevated levels of dietary calcium and magnesium, suggesting that these ions make a major contribution to Mn homeostasis in flies (Roth et al., 2013; Balachandran et al., 2020; Nyarko-Danquah et al., 2020; Kim et al., 2022). These data also point to the importance of assessing total dietary metals when developing models of Mn toxicity.

The primary differences between the diets were the use of molasses rather than sucrose as a major carbohydrate source and using deionized water to make the cornmeal diets (Table 1). Molasses is a biproduct of refining sugarcane, and likely accounts for much of the extra dietary metals (see Supplementary file 1 for manufacturer’s Nutrition fact sheet). Conversely, using deionized water appears to have been instrumental in establishing a diet with very low levels of dietary metals, and a robust response to Mn. This also provided a mechanism to easily modify the level of divalent metals added to the food, and thus our initial identification of calcium and magnesium as major players in regulating Mn toxicity. While these two metals are clearly important, we cannot rule out the possibility that the diets have additional bioenergetic affects that indirectly alter life span and/or Mn homeostasis. This is particularly evident when we consider the sucrose-yeast (SY) diet in which flies are totally resistant to 5 mM dietary Mn. The levels of sugar and yeast are known to modulate lifespan, alter food intake, and effect fecundity (Skorupa et al., 2008; Grandison et al., 2009a,b; Ormerod et al., 2017). Comparing carbohydrate and protein levels in the sucrose–yeast and cornmeal–sucrose–yeast diets, carbohydrate levels are about the same, assuming sucrose and cornmeal are reasonably equivalent sources (see Table 1), but the sucrose–yeast diet has about three-times more yeast than the cornmeal–sucrose–yeast diets. In other work, the addition of whey protein attenuated Mn toxicity (Mohandas et al., 2017). In support of an attenuated affect, adult flies have a shorter life span on the sucrose–yeast diet when slightly higher doses of Mn are provided (7.5 or 10 mM; KM Sparks, unpublished data). Thus, we suspect that subtle differences in general metabolism are also affecting the outcome of dietary Mn toxicity.

Several studies have linked calcium to Mn absorption. Calcium inhibits Mn absorption across the blood brain barrier, although increasing Mn levels do not inhibit Ca uptake (Crossgrove and Yokel, 2005). Interestingly, activation of store-operated Ca channels increases Mn uptake (Crossgrove and Yokel, 2005), and at least one member of this family, the Ca^2+^/Mn^2+^-ATPase, SPCA1, is known to move both calcium and Mn into the Golgi (Sepulveda et al., 2012; Kambe et al., 2016). Interestingly, depletion of Mn from the Golgi inhibits O-linked glycosylation (Kaufman et al., 1994) while high levels of Mn inhibit SPCA1, and initiates Golgi fragmentation (Sepulveda et al., 2012). Secretion of Mn from the cell is also considered a major detoxification pathway (Roth et al., 2013; He and Hu, 2012). In terms of secretory systems, X-ray fluorescent microscopy of the fly gut reveals that Mn and calcium are co-localized to a cluster of cells in the Malpighian tubules, an area that also expresses zinc transporters (Jones et al., 2015; Yin et al., 2017). As disruption of Golgi function and/or the secretory pathway could contribute to the altered life span of Mn-treated flies, future studies will need to address this issue.

Manganese may also be carried into cells by membrane transporters moving iron or zinc, including the Divalent Metal Transporter-1 (DMT1) and Ferroportin (Yin et al., 2010; Shawki et al., 2012; Deshpande et al., 2018). DMT1 has the highest affinity for non-heme iron, but it will move Mn^2+^ better than, for example zinc (Illing et al., 2012), and in the gut, calcium inhibits iron transporters in a reversible non-competitive manner (Cegarra et al., 2022). Magnesium may also be a weak inhibitor (Shawki and MacKenzie, 2010). However, other carrier systems may also be functioning, as selective removal of DMT from the gastrointestinal tract of mice profoundly alters iron absorption, but leaves Mn (and copper) levels unchanged (Shawki et al., 2015). Candidates include two members of the SLC39 family (Zip; zrt-, irt-like proteins), Zip8 and Zip14 which are expressed in the gut and move Mn as well as zinc or iron (Pinilla-Tenas et al., 2011; Wang et al., 2012; Winslow et al., 2020; Fujishiro and Kambe, 2022), and calcium levels affect Zip14 transport of iron and Mn^2+^ (Pinilla-Tenas et al., 2011). Meanwhile, a second family, Zinc transporters (ZnT; SLC30A), move zinc as well as iron and Mn from the cytosol and either out of cells or into the secretory pathway for excretion (Nishito et al., 2016; Taylor et al., 2019; 2023; Gurol et al., 2023).

CS adult survival on the different cornmeal diets demonstrated that elevating calcium and magnesium in the diet increases the fly's resistance to dietary Mn. The above discussion focuses on the possible mechanism by which Mn exerts toxic effects on lifespan and its interactions with dietary calcium and magnesium. Yet, interestingly, adult fly survival on the basic cornmeal diet (CO), which is relatively impoverished for all metal ions, is not robust and life span significantly changes even when a single metal ion species is added to the diet (Fig. 2), suggesting a complex interaction between metals, diet, and life span. Might the decline in survival on this poor diet reflect issues related to calcium homeostasis or even an alteration in the level of one of other trace metals? Answering this awaits further studies, including an analysis of how mutations in known metal transporter genes might affect viability, and other physiological processes, when flies are raised on these cornmeal diets. For example, *Drosophila* Zip and Znt transporters have been systematically studied and are associated with a variety of phenotypes, dietary uptake and use (Lye et al., 2012; 2013; Qin et al., 2013; Dechen et al., 2015; Richards et al., 2015; 2017). Testing these lines, as well as other mutant lines affecting calcium and magnesium transporters should yield insight into which transporters participate in Mn toxicity, and more generally in metal ion homeostasis. These studies will also provide insight into how putative disruptions in Mn homeostasis contribute to neurodegenerative conditions.

## MATERIALS AND METHODS

### Diets

Fly food used in this study include a traditional molasses-based diet, three derivatives of a cornmeal–sucrose–yeast diet (herein, simply cornmeal diet), and the sucrose–yeast diet commonly used in longevity studies. All ingredients, yeast, sucrose, molasses, cornmeal, and agar were purchased from Genesee Scientific and the percentage (w/v) of each ingredient is indicated in Table 1A. As preservatives, both Tegosept and propionic acid (Thermo Fisher Scientific) were added (v/v) once the food cooled to ∼65°C. Both molasses (MO) and sucrose–yeast (SY) foods were made using tap water, while deionized water was used to produce the three different cornmeal diets. The first, basic cornmeal diet contains only the dry ingredients with no additional metal ions added (herein CO for cornmeal – n**o** addition). For next cornmeal diet, designated CA, for cornmeal with additional divalent metals added (see Table 1B), and in the final cornmeal diet, calcium and magnesium levels were selectively enriched compared to CA, and designated CA+ [for an enriched (+) A diet]. All divalent metal ions were added from stock solutions; 1 M (1000×) stocks of MgSO_4_, MgCl_2_ and CaCl_2_, a 1000X stock solution of the CuSO_4_, MnCl_2_, and ZnSO_4_ trace metals combined, and a 500× stock solution of FeSO_4_, to final concentrations (v/v) listed in Table 1B. All chemicals were purchased from ThermoFisher Scientific. Typically, a 2 L batch of untreated food was made, which was then split to make manganese treated food by adding MnCl_2_ to 5 mM (v/v) using a 1 M stock solution. All food was dispensed (∼8 ml) into disposable vials, air-dried overnight, plugged and stored at 4°C. In the last set of experiments, Penicillin-Streptomycin antibiotics (Gibco) were added to final concentration of 10,000 unit Penicillin L^−1^ and 10,000 μg L^−1^ Streptomycin. All fly work and Biological and chemical waste management, including Mn treated food, handled as per IBC-22-06-4698, expires 8/2025.

### Survival curves

Wild-type Canton-S (CS) flies, propagated in the lab at room temperature for many years on molasses food, were used for all lifespan studies. CS flies were also propagated in bottles on the different cornmeal and sucrose–yeast diets to ensure they were suitable acclimated. Newly eclosed adults were collected and aged 1–5 days before being sexed under CO_2_ and sibling flies grouped and then randomly transferred to treated and untreated vials, 25 flies per vial at a ratio of three females to two males. The final combined *n*-values are included in tables associated with each figure and include actual deceased flies; the small percentage of escaped flies or flies trapped in food or cotton but alive were not included. For life span studies, treated and untreated vials were run in parallel in a 25°C incubator under 50% humidity and a 12-h:12-h light:dark cycle. Adults were transferred to new food three times a week, with the number of deceased flies recorded. As the work only uses CS flies, data was logged each day, but to avoid bias, survival data was only processed and compared once all flies were deceased. Kaplan–Meier survival curves were generated using Graph Pad Prizm (v9.5.1), which also calculates median survival (days). Both the Mantel–Cox log-rank test and the Gehan–Breslow–Wilcoxon test were used to assess statistical differences between a whole set of curves, as well as pairwise comparisons as needed (Bazzell et al., 2013; Sujkowski et al., 2020; 2022).

### Metal measurements

Approximately 1 g of solid food (O, MO, and SY) was dried over 3 days in a heated speed vac and hydrolyzed for 48 h with 1 ml ultra-pure nitric acid. The final hydrolysate was centrifuged to remove debris, and the supernatant diluted to a 2% acid solution using ultrapure water. Triplicate samples were analyzed using inductively coupled plasma mass spectrometry (ICP-MS) (Dr Johnna Birbeck, Lumigen Instrument Center, WSU), with the parts-per-billion converted to the mM concentration (Xiao, 2021).

### Data availability

All relevant data can be found within the article and its supplementary information.

## Supplementary Material

10.1242/biolopen.060204_sup1 Supplementary information

## References

[BIO060204C1] Adedara, I. A., Abolaji, A. O., Rocha, J. B. and Farombi, E. O. (2016). Diphenyl diselenide protects against mortality, locomotor deficits and oxidative stress in drosophila melanogaster model of manganese-induced neurotoxicity. *Neurochem. Res.* 41, 1430-1438. 10.1007/s11064-016-1852-x 26875733

[BIO060204C2] Balachandran, R. C., Mukhopadhyay, S., Mcbride, D., Veevers, J., Harrison, F. E., Aschner, M., Haynes, E. N. and Bowman, A. B. (2020). Brain manganese and the balance between essential roles and neurotoxicity. *J. Biol. Chem.* 295, 6312-6329. 10.1074/jbc.REV119.009453 32188696 PMC7212623

[BIO060204C3] Bazzell, B., Ginzberg, S., Healy, L. and Wessells, R. J. (2013). Dietary composition regulates Drosophila mobility and cardiac physiology. *J. Exp. Biol.* 216, 859-868. 10.1242/jeb.078758 23155082 PMC3571989

[BIO060204C4] Bonilla, E., Contreras, R., Medina-Leendertz, S., Mora, M., Villalobos, V. and Bravo, Y. (2012). Minocycline increases the life span and motor activity and decreases lipid peroxidation in manganese treated Drosophila melanogaster. *Toxicology* 294, 50-53. 10.1016/j.tox.2012.01.016 22330257

[BIO060204C5] Bonilla-Ramirez, L., Jimenez-Del-Rio, M. and Velez-Pardo, C. (2011). Acute and chronic metal exposure impairs locomotion activity in Drosophila melanogaster: a model to study Parkinsonism. *Biometals* 24, 1045-1057. 10.1007/s10534-011-9463-0 21594680

[BIO060204C6] Cegarra, L., Aguirre, P., Nunez, M. T., Gerdtzen, Z. P. and Salgado, J. C. (2022). Calcium is a noncompetitive inhibitor of DMT1 on the intestinal iron absorption process: empirical evidence and mathematical modeling analysis. *Am. J. Physiol. Cell Physiol.* 323, C1791-C1806. 10.1152/ajpcell.00411.2022 36342159

[BIO060204C7] Chib, S. and Singh, S. (2022). Manganese and related neurotoxic pathways: a potential therapeutic target in neurodegenerative diseases. *Neurotoxicol. Teratol.* 94, 107124. 10.1016/j.ntt.2022.107124 36183913

[BIO060204C8] Crossgrove, J. S. and Yokel, R. A. (2005). Manganese distribution across the blood-brain barrier. IV. Evidence for brain influx through store-operated calcium channels. *Neurotoxicology* 26, 297-307. 10.1016/j.neuro.2004.09.004 15935202

[BIO060204C9] Dechen, K., Richards, C. D., Lye, J. C., Hwang, J. E. and Burke, R. (2015). Compartmentalized zinc deficiency and toxicities caused by ZnT and Zip gene over expression result in specific phenotypes in Drosophila. *Int. J. Biochem. Cell Biol.* 60, 23-33. 10.1016/j.biocel.2014.12.017 25562517

[BIO060204C10] Deshpande, C. N., Ruwe, T. A., Shawki, A., Xin, V., Vieth, K. R., Valore, E. V., Qiao, B., Ganz, T., Nemeth, E., Mackenzie, B. et al. (2018). Calcium is an essential cofactor for metal efflux by the ferroportin transporter family. *Nat. Commun.* 9, 3075. 10.1038/s41467-018-05446-4 30082682 PMC6079014

[BIO060204C11] Fujishiro, H. and Kambe, T. (2022). Manganese transport in mammals by zinc transporter family proteins, ZNT and ZIP. *J. Pharmacol. Sci.* 148, 125-133. 10.1016/j.jphs.2021.10.011 34924116

[BIO060204C12] Grandison, R. C., Piper, M. D. and Partridge, L. (2009a). Amino-acid imbalance explains extension of lifespan by dietary restriction in Drosophila. *Nature* 462, 1061-1064. 10.1038/nature08619 19956092 PMC2798000

[BIO060204C13] Grandison, R. C., Wong, R., Bass, T. M., Partridge, L. and Piper, M. D. (2009b). Effect of a standardised dietary restriction protocol on multiple laboratory strains of Drosophila melanogaster. *PLoS One* 4, e4067. 10.1371/journal.pone.0004067 19119322 PMC2607010

[BIO060204C14] Gubert, P., Puntel, B., Lehmen, T., Fessel, J. P., Cheng, P., Bornhorst, J., Trindade, L. S., Avila, D. S., Aschner, M. and Soares, F. A. A. (2018). Metabolic effects of manganese in the nematode Caenorhabditis elegans through DAergic pathway and transcription factors activation. *Neurotoxicology* 67, 65-72. 10.1016/j.neuro.2018.04.008 29673961

[BIO060204C15] Guilarte, T. R. and Gonzales, K. K. (2015). Manganese-induced parkinsonism is not idiopathic Parkinson's disease: environmental and genetic evidence. *Toxicol. Sci.* 146, 204-212. 10.1093/toxsci/kfv099 26220508 PMC4607750

[BIO060204C16] Gurol, K. C., Aschner, M., Smith, D. R. and Mukhopadhyay, S. (2022). Role of excretion in manganese homeostasis and neurotoxicity: a historical perspective. *Am. J. Physiol. Gastrointest. Liver Physiol.* 322, G79-G92. 10.1152/ajpgi.00299.2021 34786983 PMC8714252

[BIO060204C17] Gurol, K. C., Li, D., Broberg, K. and Mukhopadhyay, S. (2023). Manganese efflux transporter SLC30A10 missense polymorphism T95I associated with liver injury retains manganese efflux activity. *Am. J. Physiol. Gastrointest. Liver Physiol.* 324, G78-G88. 10.1152/ajpgi.00213.2022 36414535 PMC9829465

[BIO060204C18] He, W. and Hu, Z. (2012). The role of the Golgi-resident SPCA Ca(2)(+)/Mn(2)(+) pump in ionic homeostasis and neural function. *Neurochem. Res.* 37, 455-468. 10.1007/s11064-011-0644-6 22083668

[BIO060204C19] Illing, A. C., Shawki, A., Cunningham, C. L. and Mackenzie, B. (2012). Substrate profile and metal-ion selectivity of human divalent metal-ion transporter-1. *J. Biol. Chem.* 287, 30485-30496. 10.1074/jbc.M112.364208 22736759 PMC3436370

[BIO060204C20] Jones, M. W., De Jonge, M. D., James, S. A. and Burke, R. (2015). Elemental mapping of the entire intact Drosophila gastrointestinal tract. *J. Biol. Inorg Chem.* 20, 979-987. 10.1007/s00775-015-1281-3 26153547

[BIO060204C21] Kambe, T., Takeda, T. A. and Nishito, Y. (2016). Activation of zinc-requiring ectoenzymes by ZnT transporters during the secretory process: biochemical and molecular aspects. *Arch. Biochem. Biophys.* 611, 37-42. 10.1016/j.abb.2016.03.035 27046342

[BIO060204C22] Kaufman, R. J., Swaroop, M. and Murtha-Riel, P. (1994). Depletion of manganese within the secretory pathway inhibits O-linked glycosylation in mammalian cells. *Biochemistry* 33, 9813-9819. 10.1021/bi00199a001 8060988

[BIO060204C23] Kim, H., Harrison, F. E., Aschner, M. and Bowman, A. B. (2022). Exposing the role of metals in neurological disorders: a focus on manganese. *Trends Mol. Med.* 28, 555-568. 10.1016/j.molmed.2022.04.011 35610122 PMC9233117

[BIO060204C24] Kornblith, E. S., Casey, S. L., Lobdell, D. T., Colledge, M. A. and Bowler, R. M. (2018). Environmental exposure to manganese in air: tremor, motor and cognitive symptom profiles. *Neurotoxicology* 64, 152-158. 10.1016/j.neuro.2017.09.012 28965701 PMC6260785

[BIO060204C25] Kreutzmann, P., Franz, C. and Schonfeld, P. (2012). Minocycline forms complexes with manganese in vitro: explaining reported beneficial effects in manganese treated Drosophila melanogaster. *Toxicology* 300, 100-101. 10.1016/j.tox.2012.04.010 22561279

[BIO060204C26] Lang, M., Wang, L., Fan, Q., Xiao, G., Wang, X., Zhong, Y. and Zhou, B. (2012). Genetic inhibition of solute-linked carrier 39 family transporter 1 ameliorates abeta pathology in a Drosophila model of Alzheimer's disease. *PLoS Genet.* 8, e1002683. 10.1371/journal.pgen.1002683 22570624 PMC3343105

[BIO060204C27] Levy, M., Elkoshi, N., Barber-Zucker, S., Hoch, E., Zarivach, R., Hershfinkel, M. and Sekler, I. (2019). Zinc transporter 10 (ZnT10)-dependent extrusion of cellular Mn(2+) is driven by an active Ca(2+)-coupled exchange. *J. Biol. Chem.* 294, 5879-5889. 10.1074/jbc.RA118.006816 30755481 PMC6463715

[BIO060204C28] Lye, J. C., Richards, C. D., Dechen, K., Paterson, D., De Jonge, M. D., Howard, D. L., Warr, C. G. and Burke, R. (2012). Systematic functional characterization of putative zinc transport genes and identification of zinc toxicosis phenotypes in Drosophila melanogaster. *J. Exp. Biol.* 215, 3254-3265. 22693027 10.1242/jeb.069260

[BIO060204C29] Lye, J. C., Richards, C. D., Dechen, K., Warr, C. G. and Burke, R. (2013). In vivo zinc toxicity phenotypes provide a sensitized background that suggests zinc transport activities for most of the Drosophila Zip and ZnT genes. *J. Biol. Inorg Chem.* 18, 323-332. 10.1007/s00775-013-0976-6 23322169

[BIO060204C30] Mackenzie, B., Shawki, A., Ghio, A. J., Stonehuerner, J. D., Zhao, L., Ghadersohi, S., Garrick, L. M. and Garrick, M. D. (2010). Calcium-channel blockers do not affect iron transport mediated by divalent metal-ion transporter-1. *Blood* 115, 4148-4149. 10.1182/blood-2010-03-274738 20489062 PMC2875091

[BIO060204C32] Mohandas, G., Rao, S. V., Muralidhara , and Rajini, P. S. (2017). Whey protein isolate enrichment attenuates manganese-induced oxidative stress and neurotoxicity in Drosophila melanogaster: relevance to Parkinson's disease. *Biomed. Pharmacother.* 95, 1596-1606. 10.1016/j.biopha.2017.09.099 28950660

[BIO060204C33] Nishito, Y., Tsuji, N., Fujishiro, H., Takeda, T. A., Yamazaki, T., Teranishi, F., Okazaki, F., Matsunaga, A., Tuschl, K., Rao, R. et al. (2016). Direct comparison of manganese detoxification/efflux proteins and molecular characterization of ZnT10 protein as a manganese transporter. *J. Biol. Chem.* 291, 14773-14787. 10.1074/jbc.M116.728014 27226609 PMC4938194

[BIO060204C34] Nyarko-Danquah, I., Pajarillo, E., Digman, A., Soliman, K. F. A., Aschner, M. and Lee, E. (2020). Manganese accumulation in the brain via various transporters and its neurotoxicity mechanisms. *Molecules* 25, 5880. 10.3390/molecules25245880 33322668 PMC7763224

[BIO060204C35] Oboh, G., Ogunsuyi, O. B., Awonyemi, O. I. and Atoki, V. A. (2018). Effect of alkaloid extract from african jointfir (gnetum africanum) leaves on manganese-induced toxicity in drosophila melanogaster. *Oxid. Med. Cell Longev.* 2018, 8952646. 10.1155/2018/8952646 30693067 PMC6332884

[BIO060204C36] O'Neal, S. L. and Zheng, W. (2015). Manganese toxicity upon overexposure: a decade in review. *Curr. Environ. Health Rep.* 2, 315-328. 10.1007/s40572-015-0056-x 26231508 PMC4545267

[BIO060204C37] Ormerod, K. G., Lepine, O. K., Abbineni, P. S., Bridgeman, J. M., Coorssen, J. R., Mercier, A. J. and Tattersall, G. J. (2017). Drosophila development, physiology, behavior, and lifespan are influenced by altered dietary composition. *Fly* 11, 153-170. 10.1080/19336934.2017.1304331 28277941 PMC5552271

[BIO060204C38] Pankau, C. and Cooper, R. L. (2022). Molecular physiology of manganese in insects. *Curr. Opin. Insect. Sci.* 51, 100886. 10.1016/j.cois.2022.100886 35278758

[BIO060204C39] Pankau, C., Nadolski, J., Tanner, H., Cryer, C., Di Girolamo, J., Haddad, C., Lanning, M., Miller, M., Neely, D., Wilson, R. et al. (2022). Examining the effect of manganese on physiological processes: invertebrate models. *Comp. Biochem. Physiol. C Toxicol. Pharmacol.* 251, 109209. 10.1016/j.cbpc.2021.109209 34628058 PMC8922992

[BIO060204C40] Pfalzer, A. C. and Bowman, A. B. (2017). Relationships between essential manganese biology and manganese toxicity in neurological disease. *Curr. Environ. Health Rep.* 4, 223-228. 10.1007/s40572-017-0136-1 28417441 PMC5515274

[BIO060204C41] Pfalzer, A. C., Wilcox, J. M., Codreanu, S. G., Totten, M., Bichell, T. J. V., Halbesma, T., Umashanker, P., Yang, K. L., Parmalee, N. L., Sherrod, S. D. et al. (2020). Huntington's disease genotype suppresses global manganese-responsive processes in pre-manifest and manifest YAC128 mice. *Metallomics* 12, 1118-1130. 10.1039/d0mt00081g 32421118 PMC7773276

[BIO060204C42] Pinilla-Tenas, J. J., Sparkman, B. K., Shawki, A., Illing, A. C., Mitchell, C. J., Zhao, N., Liuzzi, J. P., Cousins, R. J., Knutson, M. D. and Mackenzie, B. (2011). Zip14 is a complex broad-scope metal-ion transporter whose functional properties support roles in the cellular uptake of zinc and nontransferrin-bound iron. *Am. J. Physiol. Cell Physiol.* 301, C862-C871. 10.1152/ajpcell.00479.2010 21653899 PMC3191563

[BIO060204C43] Power, J. H., Barnes, O. L. and Chegini, F. (2017). Lewy bodies and the mechanisms of neuronal cell death in parkinson's disease and dementia with lewy bodies. *Brain Pathol.* 27, 3-12. 10.1111/bpa.12344 26667592 PMC8029402

[BIO060204C44] Qin, Q., Wang, X. and Zhou, B. (2013). Functional studies of Drosophila zinc transporters reveal the mechanism for dietary zinc absorption and regulation. *BMC Biol.* 11, 101. 10.1186/1741-7007-11-101 24063361 PMC4015762

[BIO060204C45] Richards, C. D., Warr, C. G. and Burke, R. (2015). A role for dZIP89B in Drosophila dietary zinc uptake reveals additional complexity in the zinc absorption process. *Int. J. Biochem. Cell Biol.* 69, 11-19. 10.1016/j.biocel.2015.10.004 26545796

[BIO060204C46] Richards, C. D., Warr, C. G. and Burke, R. (2017). A role for the Drosophila zinc transporter Zip88E in protecting against dietary zinc toxicity. *PLoS One* 12, e0181237. 10.1371/journal.pone.0181237 28704512 PMC5509326

[BIO060204C47] Roth, J., Ponzoni, S. and Aschner, M. (2013). Manganese homeostasis and transport. *Met. Ions Life Sci.* 12, 169-201. 10.1007/978-94-007-5561-1_6 23595673 PMC6542352

[BIO060204C49] Sepulveda, M. R., Wuytack, F. and Mata, A. M. (2012). High levels of Mn(2)(+) inhibit secretory pathway Ca(2)(+)/Mn(2)(+)-ATPase (SPCA) activity and cause Golgi fragmentation in neurons and glia. *J. Neurochem.* 123, 824-836. 10.1111/j.1471-4159.2012.07888.x 22845487

[BIO060204C50] Shawki, A. and Mackenzie, B. (2010). Interaction of calcium with the human divalent metal-ion transporter-1. *Biochem. Biophys. Res. Commun.* 393, 471-475. 10.1016/j.bbrc.2010.02.025 20152801 PMC2838957

[BIO060204C51] Shawki, A., Knight, P. B., Maliken, B. D., Niespodzany, E. J. and Mackenzie, B. (2012). H(+)-coupled divalent metal-ion transporter-1: functional properties, physiological roles and therapeutics. *Curr. Top. Membr.* 70, 169-214. 10.1016/B978-0-12-394316-3.00005-3 23177986 PMC7027397

[BIO060204C52] Shawki, A., Anthony, S. R., Nose, Y., Engevik, M. A., Niespodzany, E. J., Barrientos, T., Ohrvik, H., Worrell, R. T., Thiele, D. J. and Mackenzie, B. (2015). Intestinal DMT1 is critical for iron absorption in the mouse but is not required for the absorption of copper or manganese. *Am. J. Physiol. Gastrointest. Liver Physiol.* 309, G635-G647. 10.1152/ajpgi.00160.2015 26294671 PMC4609933

[BIO060204C53] Silva, N. C., Poetini, M. R., Bianchini, M. C., Almeida, F. P., Dahle, M. M. M., Araujo, S. M., Bortolotto, V. C., Musachio, E. A. S., Ramborger, B. P., Novo, D. R. et al. (2021). Protective effect of gamma-oryzanol against manganese-induced toxicity in Drosophila melanogaster. *Environ. Sci. Pollut. Res. Int.* 28, 17519-17531. 10.1007/s11356-020-11848-z 33403631

[BIO060204C54] Skorupa, D. A., Dervisefendic, A., Zwiener, J. and Pletcher, S. D. (2008). Dietary composition specifies consumption, obesity, and lifespan in Drosophila melanogaster. *Aging Cell* 7, 478-490. 10.1111/j.1474-9726.2008.00400.x 18485125 PMC2574586

[BIO060204C55] Sujkowski, A., Gretzinger, A., Soave, N., Todi, S. V. and Wessells, R. (2020). Alpha- and beta-adrenergic octopamine receptors in muscle and heart are required for Drosophila exercise adaptations. *PLoS Genet.* 16, e1008778. 10.1371/journal.pgen.1008778 32579604 PMC7351206

[BIO060204C56] Sujkowski, A., Richardson, K., Prifti, M. V., Wessells, R. J. and Todi, S. V. (2022). Endurance exercise ameliorates phenotypes in Drosophila models of spinocerebellar ataxias. *Elife* 11, e75389. 10.7554/eLife.75389 35170431 PMC8871352

[BIO060204C57] Taylor, C. A., Hutchens, S., Liu, C., Jursa, T., Shawlot, W., Aschner, M., Smith, D. R. and Mukhopadhyay, S. (2019). SLC30A10 transporter in the digestive system regulates brain manganese under basal conditions while brain SLC30A10 protects against neurotoxicity. *J. Biol. Chem.* 294, 1860-1876. 10.1074/jbc.RA118.005628 30559290 PMC6369308

[BIO060204C58] Taylor, C. A., Tuschl, K., Nicolai, M. M., Bornhorst, J., Gubert, P., Varao, A. M., Aschner, M., Smith, D. R. and Mukhopadhyay, S. (2020). Maintaining translational relevance in animal models of manganese neurotoxicity. *J. Nutr.* 150, 1360-1369. 10.1093/jn/nxaa066 32211802 PMC7269748

[BIO060204C59] Taylor, C. A., Grant, S. M., Jursa, T., Melkote, A., Fulthorpe, R., Aschner, M., Smith, D. R., Gonzales, R. A. and Mukhopadhyay, S. (2023). SLC30A10 manganese transporter in the brain protects against deficits in motor function and dopaminergic neurotransmission under physiological conditions. *Metallomics* 15, mfad021. 10.1093/mtomcs/mfad021 36990693 PMC10103839

[BIO060204C60] Tinkov, A. A., Paoliello, M. M. B., Mazilina, A. N., Skalny, A. V., Martins, A. C., Voskresenskaya, O. N., Aaseth, J., Santamaria, A., Notova, S. V., Tsatsakis, A. et al. (2021). Molecular targets of manganese-induced neurotoxicity: a five-year update. *Int. J. Mol. Sci.* 22, 4646. 10.3390/ijms22094646 33925013 PMC8124173

[BIO060204C61] Wang, X., Wu, Y. and Zhou, B. (2009). Dietary zinc absorption is mediated by ZnT1 in Drosophila melanogaster. *FASEB J.* 23, 2650-2661. 10.1096/fj.08-126649 19325039

[BIO060204C62] Wang, C.-Y., Jenkitkasemwong, S., Duarte, S., Sparkman, B. K., Shawki, A., Mackenzie, B. and Knutson, M. D. (2012). ZIP8 is an iron and zinc transporter whose cell-surface expression is up-regulated by cellular iron loading. *J. Biol. Chem.* 287, 34032-34043. 10.1074/jbc.M112.367284 22898811 PMC3464513

[BIO060204C63] Wang, Z., Li, X. and Zhou, B. (2020). Drosophila ZnT1 is essential in the intestine for dietary zinc absorption. *Biochem. Biophys. Res. Commun.* 533, 1004-1011. 10.1016/j.bbrc.2020.09.077 33012507

[BIO060204C64] Winslow, J. W. W., Limesand, K. H. and Zhao, N. (2020). The functions of ZIP8, ZIP14, and ZnT10 in the regulation of systemic manganese homeostasis. *Int. J. Mol. Sci.* 21, 3304. 10.3390/ijms21093304 32392784 PMC7246657

[BIO060204C65] Xiao, G. (2021). Determination of metal content in drosophila melanogaster during metal exposure. *Methods Mol. Biol.* 2326, 327-337. 10.1007/978-1-0716-1514-0_24 34097280

[BIO060204C66] Yin, Z., Jiang, H., Lee, E. S., Ni, M., Erikson, K. M., Milatovic, D., Bowman, A. B. and Aschner, M. (2010). Ferroportin is a manganese-responsive protein that decreases manganese cytotoxicity and accumulation. *J. Neurochem.* 112, 1190-1198. 10.1111/j.1471-4159.2009.06534.x 20002294 PMC2819584

[BIO060204C67] Yin, S., Qin, Q. and Zhou, B. (2017). Functional studies of Drosophila zinc transporters reveal the mechanism for zinc excretion in Malpighian tubules. *BMC Biol.* 15, 12. 10.1186/s12915-017-0355-9 28196538 PMC5309981

[BIO060204C68] Zogzas, C. E. and Mukhopadhyay, S. (2018). Putative metal binding site in the transmembrane domain of the manganese transporter SLC30A10 is different from that of related zinc transporters. *Metallomics* 10, 1053-1064. 10.1039/C8MT00115D 29989630 PMC6093791

