# Supplementary material for: Divalent metal content in diet affects severity of manganese toxicity in Drosophila
Source: Biol Open. 2024 Jan 5;13(1):bio060204. doi: 10.1242/bio.060204 (PMC10810561; doi:10.1242/bio.060204)
Supplement: Supplementary information [file biolopen-13-060204-s1.pdf]

Molasses – Golden A, Ingredient

Sweet Harvest Foods

PRODUCT DESCRIPTION:
Dark colored brown natural molasses blend with the flavor and aroma of fine imported unsulphured molasses. This syrup possesses the natural flavor and aroma of a premium cane molasses.

APPLICATIONS:
Breads, rolls, cookies, syrups, toppings, sauces, BBQ, and confections.

INGREDIENT DECLARATION:
Cane Molasses

COUNTRY OF ORIGIN:
Product of USA

ALLERGEN & SENSITIVE COMPONENT:
None

SAFETY DATA SHEET:
Not required

HACCP PLAN:
On file for customer review

KOSHER CERTIFICATION:
1. Union of Orthodox Jewish Congregations of America.
2. The OU Kosher symbol shall appear on each product container. Ⓤ

SHIPPING:
1. All trucks used for transporting the material must be clean, dry and free from conditions that could result in contamination of the raw material.
2. Less than truckload (LTL) quantities must not ship with product that may be harmful or deleterious of nature.
3. A packing slip must be included with each shipment.

PHYSICAL PROPERTIES:

Weight/Gallon	11.7 Pounds

PRODUCT ANALYSIS:

PHYSICAL	TYPICAL RANGE	ANALYSIS METHOD
Double Diluted Brix (1:1) (1)	> 79.0	170-CHM-12 (2)
pH (1)	4.8 – 6.5	170-CHM-31 (2)
Reducing Sugars % Wet (1)	28.0 – 43.0	170-CHM-17 (2)
Sucrose % Wet (1)	28.0 – 43.0	170-CHM-53 (2)
Total Sugars % Wet (1)	> 66.0	170-CHM-53 (2)

Notes:
(1) Result source: Supplier specification.
(2) Supplier Method.

PACKAGE LABELING

Manufacturer's Name & Location	Product Type & Container Type
ERP Product Code & Bar Code	Serial Number
Lot Number	Country of Origin
Kosher Symbol	Date of Mfg & Best Use By Date

CODE DATE/LOT INFORMATION:
Sweet Harvest Foods molasses lots are identified as follows:

Example:	LOT17110300
Year:	17
Month:	11
Randomly Generated Number:	0300

Biology Open • Supplementary information

Sweet Harvest Foods

Molasses – Golden A, Ingredient

SHELF-LIFE GUIDELINES:

Guidelines when stored at proper temperatures:

Best Used By	9 Months

STORAGE:

Store in a cool, clean, dry area; ambient temperature 75ºF +/- 5ºF and not above 50% relative humidity.

NUTRITIONAL INFORMATION:

Based on 100g

Calories	324.5
Protein	1.13 g
Carbohydrates	80.27 g
Dietary Fiber	0.0 g
Total Sugars	79.58 g
Added Sugars	79.58 g
Fat	0.02 g
Saturated Fat	0.0 g
Trans Fat	0.0 g
Cholesterol	0.00 mg
Water	21.22 g
Calcium	61.52 mg
Iron	1.70 mg
Sodium	17.00 mg

PACKAGING:

PRODUCT #	SIZES	CONTAINER	PALLET
F522	60 lbs.	HDPE 20L/5G Pail w/ Locking HDPE Lid	24 or 36
F524	3,000 lbs.	Plastic 275G Returnable Tote IBC w/ Steel Cage	N/A
F526	3,000 lbs.	Plastic 275G Returnable Tote IBC w/ Steel Cage	N/A
C113	40,000 lbs.	Food Grade Tanker	N/A

Biology Open • Supplementary information
